# Impacts of Arctic Shrubs on Root Traits and Belowground Nutrient Cycles Across a Northern Alaskan Climate Gradient

**DOI:** 10.3389/fpls.2020.588098

**Published:** 2020-12-11

**Authors:** Weile Chen, Ken D. Tape, Eugénie S. Euskirchen, Shuang Liang, Adriano Matos, Jonathan Greenberg, Jennifer M. Fraterrigo

**Affiliations:** ^1^Department of Natural Resources and Environmental Sciences, University of Illinois at Urbana-Champaign, Urbana, IL, United States; ^2^College of Life Sciences, Zhejiang University, Hangzhou, China; ^3^Geophysical Institute, University of Alaska Fairbanks, Fairbanks, AK, United States; ^4^Institute of Arctic Biology, University of Alaska Fairbanks, Fairbanks, AK, United States; ^5^Department of Natural Resources and Environmental Science, University of Nevada, Reno, Reno, NV, United States

**Keywords:** arctic shrub expansion, ectomycorrhizal fungi, plant-soil interactions, rooting depth, root economics spectrum, trait-based approach, soil nitrogen

## Abstract

Deciduous shrubs are expanding across the graminoid-dominated nutrient-poor arctic tundra. Absorptive root traits of shrubs are key determinants of nutrient acquisition strategy from tundra soils, but the variations of shrub root traits within and among common shrub genera across the arctic climatic gradient are not well resolved. Consequently, the impacts of arctic shrub expansion on belowground nutrient cycling remain largely unclear. Here, we collected roots from 170 plots of three commonly distributed shrub genera (*Alnus*, *Betula*, and *Salix*) and a widespread sedge (*Eriophorum vaginatum*) along a climatic gradient in northern Alaska. Absorptive root traits that are relevant to the strategy of plant nutrient acquisition were determined. The influence of aboveground dominant vegetation cover on the standing root biomass, root productivity, vertical rooting profile, as well as the soil nitrogen (N) pool in the active soil layer was examined. We found consistent root trait variation among arctic plant genera along the sampling transect. *Alnus* and *Betula* had relatively thicker and less branched, but more frequently ectomycorrhizal colonized absorptive roots than *Salix*, suggesting complementarity between root efficiency and ectomycorrhizal dependence among the co-existing shrubs. Shrub-dominated plots tended to have more productive absorptive roots than sedge-dominated plots. At the northern sites, deep absorptive roots (>20 cm depth) were more frequent in birch-dominated plots. We also found shrub roots extensively proliferated into the adjacent sedge-dominated plots. The soil N pool in the active layer generally decreased from south to north but did not vary among plots dominated by different shrub or sedge genera. Our results reveal diverse nutrient acquisition strategies and belowground impacts among different arctic shrubs, suggesting that further identifying the specific shrub genera in the tundra landscape will ultimately provide better predictions of belowground dynamics across the changing arctic.

## Introduction

Plant roots are essential for nutrient acquisition across global biomes. The diversity of root characteristics (“root traits,” e.g., root thickness) among different plant species indicates varying nutrient acquisition strategies (Fitter, 1982; Bardgett et al., 2014; Ma et al., 2018). Roots of terrestrial plants also frequently interact with mutualistic mycorrhizal fungi that can increase the absorptive surface and, in some cases, mobilize nutrients trapped in soil organic matter (Chapman et al., 2006; Smith and Read, 2008; Shah et al., 2016). Studies in tropical and temperate biomes suggest that plant species that mainly rely on the root pathway in nutrient acquisition tend to form thinner roots with higher nitrogen (N) concentrations, which enable them to more rapidly leverage resources for soil exploration. In contrast, species that primarily acquire nutrients through the mycorrhizal fungal pathway may form roots with lower N concentrations but with larger root diameter that favors mycorrhizal fungal colonization (Bergmann et al., 2020). Variations of root traits can also exist among the common arctic plants, but whether the complementary variation in root vs. mycorrhizal fungal dependence of nutrient acquisition exists among arctic plants remains to be tested (Iversen et al., 2015).

In part of the sedge-dominated arctic tundra, shrub species are expanding their aboveground cover during the past several decades (Sturm et al., 2001b). Shrub and sedge roots may have developed divergent root traits and strategies to meet their nutrient requirement in the nutrient-poor arctic tundra. For instance, the widespread sedge *Eriophorum vaginatum* has thick, unbranched and non-mycorrhizal roots. *Eriophorum* roots can effectively absorb simple forms of organic nutrients because aerenchyma in *Eriophorum* roots can facilitate nutrient acquisition and transportation (e.g., glycine, Chapin et al., 1993; McKane et al., 2002). In contrast, the common arctic deciduous shrubs (e.g., *Alnus*, *Betula*, and *Salix*) often form thin, highly branched, and ectomycorrhizal roots, indicating high capacity in nutrient acquisition (Iversen et al., 2015; Weemstra et al., 2016). In addition to the influence of plant identity on root traits, local climatic and edaphic conditions can also drive variation in root traits and nutrient acquisition strategies along large-scale environmental gradients (Zadworny et al., 2016). Moreover, because shrubs are among the tundra plant species most responsive to environmental change (Bret-Harte et al., 2001, 2002), and because of the various adaptations of different shrub genera along the climatic and edaphic gradients (Young et al., 2016; Rinas et al., 2017), the interspecific variations of root traits among arctic plants may also vary along environmental gradients. However, unlike the widely observed variations in leaf traits among arctic plants (Bjorkman et al., 2018; Thomas et al., 2020), the spatial patterns of root trait variation across broad ranges and the underlying biotic and abiotic factors that drive root trait variation in the arctic are not well understood (Butler et al., 2017).

An additional consequence of arctic shrub expansion in the sedge-dominated tundra is the potential changes in belowground root biomass, vertical root distribution, and root productivity. Previous studies showed that sedges tend to be more deeply rooted in the soil profile than shrubs (Iversen et al., 2015; Wang et al., 2016; Hewitt et al., 2019b), whereas accumulative root biomass in the active layer might be greater under shrubs than sedges since shrubs are usually taller and more productive than sedges (Wang et al., 2016; Blume−Werry et al., 2019). The root biomass and patterns of root vertical distribution might vary among different shrub genera because of their diverse allocation strategies and physical and biological impacts on the soil environments, but relevant studies across a large climatic gradient remain rare in the arctic tundra.

The paucity of root data in the Arctic has been exacerbated by inadequate characterization of roots with absorptive function. Distal roots (lower-order roots) of woody plants are often non-woody and responsible for resource acquisition, while higher-order roots are woody and responsible for transport and storage (Guo et al., 2008; McCormack et al., 2015). Unfortunately, functional variations within the root systems of arctic plants are usually overlooked, and existing classifications that do not entirely represent the absorptive part of the root system (e.g., “fine roots” defined as roots with diameter <2 mm) have been predominately used in root studies of arctic or sub-arctic plants (Björk et al., 2007; Freschet et al., 2010a; Hewitt et al., 2019b). A systematic examination of absorptive roots that actively engage in resource acquisition of arctic plant species has not been conducted and is needed to better understand nutrient acquisition strategies and nutrient dynamics in arctic tundra ecosystems.

Together, the potential variations in absorptive root traits and biomass under different vegetation cover may lead to differences in how nutrients are acquired by arctic plants and how nutrients are cycled in the ecosystem (Weintraub and Schimel, 2005). The woody detritus derived from shrubs are high in carbon (C): N and decompose slowly in arctic soils (Hobbie, 1996), which may lead to higher amount of soil C stored per unit soil N under the shrubs than the herbaceous plants. In addition, the potential widespread association of ectomycorrhizal fungi with arctic shrubs may further contribute to changes in N cycling, as ectomycorrhizas can also lead to greater storage of soil C per unit N (Averill et al., 2014). However, to what extent the woody and ectomycorrhizal shrubs have influenced the cycling and storage of soil N in the arctic tundra, and whether the changes in soil N varies among shrub genera remains unclear. In addition, the nitrogen-fixing capacity of alder may introduce additional complexity to the soil N cycles in the arctic tundra (Mitchell and Ruess, 2009). Given that large pools of soil organic N resulting from slow decomposition are stored in this region (Post et al., 1985), it is important to resolve the belowground data gap of root traits, root biomass, and mycorrhizal associations to better predict N dynamics in the arctic tundra.

In this study, we examined multiple traits of absorptive roots relevant to the nutrient acquisition of common sedge and shrub genera in Northern Alaska. Root samples were collected along a latitudinal gradient to study the influence of environmental conditions on root traits. We also quantified the biomass of absorptive roots and mapped their vertical distributions in the active soil layer across the latitudinal gradient. Finally, we examined differences in the soil N pool and C: N among shrub-dominated and sedge-dominated plots. We hypothesize that (1) Absorptive root traits differ among arctic plants and vary along the climatic gradient; (2) Absorptive roots and ectomycorrhizal fungal colonization are functionally complementary among the arctic shrubs; (3) Both plant genera and local climate can influence the belowground absorptive root biomass and productivity, soil N pool size, and soil C to N ratio.

## Materials and Methods

### Study Sites

**FIGURE 1:**
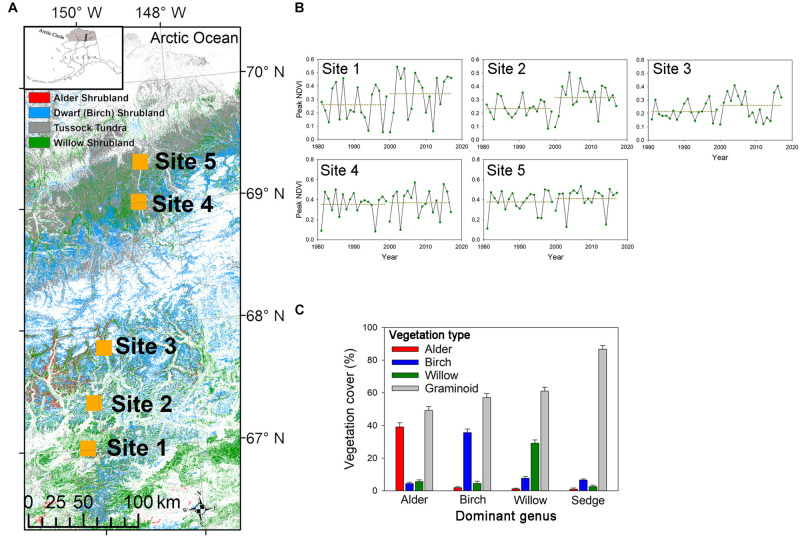
**(A)** The locations of five sampling sites along a latitudinal gradient in Northern Alaska. Background color represents the land cover of deciduous shrublands and tussock tundra (Source: LANDFIRE 2014 LF 1.4.0, https://www.landfire.gov). The upper left inset shows the arctic region of Alaska (gray-shaded) and our sampling transect (black-shaded). **(B)** Time series of peak AVHRR NDVI data from 1981 to 2017. Points represent the peak monthly NDVI from May to September each year. Two dash lines represent averaged peak NDVI in the earlier years (1981–1999) and later years (2000–2017). At the two southern sites, averaged peak NDVI significantly increased from earlier to later years (*P* = 0.03 for both sites). **(C)** Vegetation cover of the sampling plots. In shrub-dominated plots, the target shrub (alder, birch, or willow) was that which showed the largest cover among the three shrub genera; while in sedge-dominated plots, shrub cover was almost negligible, and *Eriophorum vaginatum* were the major graminoid species in the plot.

In June and July of 2017, we collected roots and soil from five sampling sites along a latitudinal gradient in Northern Alaska (67.0°N–69.3°N; Figure 1A), encompassing a wide range of summer soil temperature (5.2°C–8.9°C; Table 1) and climatic variables (Supplementary Table 1). We analyzed a 37-year time series of satellite data to examine the trend of vegetation greenness change in the five sampling sites (Figure 1B). The normalized difference vegetation index (NDVI) data obtained by the Advanced Very High-Resolution Radiometer (AVHRR) was retrieved from Google Earth Engine’s public catalog using the platform’s JavaScript API. We calculated the monthly median values for any datasets that collected > 1 image per month, resulting, for every dataset, one single value per month across the available range of dates. We selected the highest area-averaged monthly NDVI each year to represent the peak vegetation greenness in each sampling area. Analysis of satellite imagery indicated an increase of vegetation greenness at the two southern sites (Figure 1B), probably due to the expansion of shrub abundance in the Alaskan tundra areas (Jia et al., 2003; Ju and Masek, 2016).

**TABLE 1:** Sampling site information.

**Site**	**Latitude (°N)**	**Longitude (°W)**	**Elevation (m.a.s.l)**	**Soil temperature (°C)**	**Mean thaw depth (organic + mineral layer, cm)**
S1	67.01	150.29	338	8.9	31 (27 + 4)
S2	67.38	150.10	414	7.9	24 (21 + 3)
S3	67.83	149.82	563	6.3	27 (20 + 7)
S4	69.01	148.83	369	6.0	21 (16 + 5)
S5	69.34	148.72	227	5.2	18 (15 + 3)

*Mean thaw depth was the site-average depth of the soil cores sampled at each plot in late June – early July 2017. Soil temperature of each site was measured at 10 cm depth in late July 2018.*

At each sampling site, we identified species from three expanding deciduous shrub genera *Alnus* (*A. viridis ssp. fruticosa*), *Betula* (*B. nana*), and *Salix* (*S. glauca, S. pulchra, S. arbuscules*, or *S. richardsonii*), respectively, alder, birch, and willow. The *Salix* species sampled in each site are shown in Supplementary Table 2. For each selected shrub genera, we established nine plots in each site. We also established seven tussock sedge plots in each site that were dominated by *E. vaginatum* and close to the shrub plots, resulting in 170 sampled plots across the Northern Alaskan sampling transect (Figure 1A). All plots were 1-m-diameter circles surrounding the stem of the selected shrub individual or the tussock sedge. Percentages of vegetation cover of the three shrubs and graminoids were estimated for each plot. In shrub-dominated plots, the target shrub was that which showed the largest cover among the three shrub genera; while in sedge-dominated plots, shrub cover was almost negligible (Figure 1C).

### Root Trait Measurements

We harvested root samples from the target plant for trait measurements in the upper 20 cm of soil in each plot to avoid confounding by soil depth. In shrub-dominated plots, we excavated intact root branches linked to the target shrub individual. At least six intact root branches, which included the 1st to 6th or higher orders at a stream-based ordering system with the first order being the most distal roots (*sensu* Guo et al., 2008) were carefully cut and stored in coolers before returning to the lab. In sedge-dominated plots, the live intact roots (light color and dense tissue) that connected to the rhizome of *E. vaginatum* were also cut and stored. In addition, at least 10 sun-leaves of the target plant from which roots were harvested were collected. All root and leaf samples were kept at -20°C in the lab until processing.

In the lab, roots were carefully rinsed with deionized water to remove soil particles. We considered the first three root orders as absorptive roots for shrubs (*sensu* McCormack et al., 2015). Because *E. vaginatum* roots do not have branching architecture, all individual *E. vaginatum* root segments were considered absorptive roots, but only intact *E. vaginatum* root segments were selected for later trait analysis. A subset of approximately fifteen absorptive roots from each plot was selected for scanning on a desktop scanner. All scanned root images were processed with WinRHIZO (Regent Instruments, Quebec, Canada) to determine the average root diameter. Root architectures of shrub roots were also determined from scanned images. The number of 1st, 2nd, and 3rd order roots was counted in each image, and branching ratios were calculated as the number of 1st-order roots per each 2nd-order root (BR1-2) and the number of 2nd-order roots per each 3rd-order root (BR2-3). The scanned roots, together with the leaves, were oven-dried at 55°C for 72 h and ground to a fine powder. Root and leaf tissue nitrogen concentration ([N]), as well as N isotopic signature δ^15^N, was determined using a Costech 4010 CHNSO Elemental Analyzer (Costech Analytical Technologies Inc., Valencia, CA, United States) interfaced with an isotope ratio mass spectrometer (Thermo Fisher Delta V Advantage, Fisher Scientific) at the University of Illinois. Another subset of fresh shrub root sample was preserved in 70% (v/v) ethanol for later determination of the percentage of root tips that were colonized by ectomycorrhizal fungi by inspection of root tip morphology, color, architecture, and anatomy.

### Root Biomass and Vertical Distribution

Within each plot, an intact soil core (moss + organic + mineral layer) was manually sampled using a 7.6-cm-diameter soil corer with a post driver until reaching the permafrost layer. The intact soil core was wrapped using aluminum foil in the field. Soil cores were stored in coolers until transferred to the -20°C freezers. The depth of the resulting hole was measured. In the lab, soil cores were carefully placed on the bench and total length were measured. The moss layer (if any) usually rapidly reshaped and we measured the depth of the moss layer before it was removed from the soil core. The remaining soil core was separated into the organic and mineral layers based on the visible differences in organic matter content. Lengths of the organic and mineral layers were measured. The soil core was then sliced into two halves, with one half reserved for later soil analysis. The other half was used to quantify absorptive root biomass. This half soil core was cut into 2-cm thick pieces, labeled by position within the soil core (i.e., core depth) and converted to field depth to adjust for compression of the organic layer during sampl]ing:

$$\begin{array}{c} D_{f\,i\,e\,l\,d-o\,r\,g\,a\,n\,i\,c}=D_{c\,o\,r\,e}\times \left(L_{h\,o\,l\,e}-L_{m\,o\,s\,s}-L_{m\,i\,n\,e\,r\,a\,l}\right)/\\ \left(L_{c\,o\,r\,e}-L_{m\,o\,s\,s}-L_{m\,i\,n\,e\,r\,a\,l}\right),\\ D_{f\,i\,e\,l\,d-m\,i\,n\,e\,r\,a\,l}=D_{c\,o\,r\,e}+\left(L_{h\,o\,l\,e}-L_{m\,o\,s\,s}-L_{m\,i\,n\,e\,r\,a\,l}-L_{o\,r\,g\,a\,n\,i\,c}\right) \end{array}$$

Where *D_*field*__–org__*anic*_* and *D_*field–min*__*eral*_* was the depth of an organic or mineral soil-piece in the field (excluding moss layer), and *D*_*core*_ was its depth measured in the lab. *L*_*hole*_ was the total length of the hole resulting from soil coring to the permafrost, and *L*_*core*_ was the length of the intact cores measured in the lab (moss layer + organic layer + mineral layer). *L*_*moss*_ was the length of the moss layer (if any), *L*_*mineral*_ and *L*_*organic*_ was the length of the mineral and organic layer measured in the lab.

After the soil cores were divided into different depth increments, all roots from each depth increment were carefully picked and rinsed. Because roots of different plant species inside and outside the plot could intermix at fine scales, and because of the difficulty in differentiating roots among species, the root biomass at different depths represents root vertical distribution at a plant-community level. In particular, only live roots (based on root color, surface structure, and tissue density) were collected. Intact roots were further separated into absorptive vs. non-absorptive (woody) roots based on root orders. For fragmentary roots, root segments that were rigid and showed woody anatomical structure on the intersecting surface were identified as woody roots and the remaining were non-woody absorptive roots. Both absorptive and non-absorptive roots were oven-dried at 55°C for 72 h and weighed. The depth increment of each sample and the inner core radius was used to calculate root biomass density at different soil depths. In total, we collected and measured root biomass from 1172 pieces of soil core sections.

### Root Ingrowth Experiment

We established a root ingrowth experiment to estimate community-level root productivity in both shrub and sedge dominated plots. Four ingrowth containers were installed at each plot in late June and early July 2017. Ingrowth containers were made from a plastic mesh tube with 3.5-cm diameter, 10-cm length, and 4-mm aperture (Chen et al., 2016). Four random locations were selected within each plot, and a bread-knife was used to remove a 3.5-cm diameter, 10-cm deep soil core. The initial soil cores were collected for later root biomass measurements. Nodules were also identified from initial soil cores collected in alder-dominated plots and weighed (Supplementary Figure 1). Ingrowth containers were then inserted into the resulting holes and filled with root-free pure sand. We used sand instead of natural soil because of the extreme difficulty in identifying and removing roots from a large amount of high organic-content soil to fill all the ingrowth containers in five remote sites. The sand was supplemented by adding 2 *g* of finely-cut oven-dried green leaves of the same genera (birch, willow, alder, and sedge) in the first one-third depth of the container as slow-release nutrient source bait for root growth. The leaves that were used were harvested near Fairbanks, Alaska about 2 months prior to the ingrowth experiment. Two ingrowth containers in each plot were harvested in September 2017 to check root ingrowth, and the remaining two containers were harvested in July 2018. Roots from the initial soil cores collected in June-July 2017, as well as from ingrowth cores harvested in July 2018 were fully cleaned, dried and weighed. Annual root productivity was determined in both absolute and relative manners: absolute root productivity was the biomass in the ingrowth cores harvested in July 2018; and relative root production was the ratio of absolute root production to the averaged initial root biomass. Although root growth in the media of sand + leaf litter may not fully represent the growth in natural soils, this measurement of root growth provides a useful comparison across different plots.

### Soil Assay

Large roots and rocks were removed from the remaining half soil cores (see section “*Root biomass and vertical distribution*”). We then determined total fresh weight and gravimetric moisture (105°C for 48 h) to obtain the total dry weight of soil in the organic and mineral layers of each soil core. In addition, the particulate organic matter (POM, >53 μm) pool, which represents the partially decomposed plant material in the soil organic matter pool, was extracted for the organic soil layer using a size fractionation procedure (Cambardella and Elliott, 1994) as modified by Bradford et al. (2008). Briefly, about 10 *g* of air-dried, 2 mm sieved soil was placed into a 30-ml-plastic bottle with a 53-μm mesh lid. These small containers were shaken in a larger container filled with a 5% sodium hexametaphosphate solution for 1 h and rinsed with deionized water for ten 10-min intervals (changing out the water each time). After washing, the POM fraction remaining in each small container was dried (105°C for 48 h) and weighed.

The C and N concentration, as well as δ^15^N values of bulk soil and the POM component, were determined using the aforementioned elemental analyzer and mass spectrometer. To calculate the area-based total C and N pool size in soil above the permafrost, soil C and N concentrations of either the organic or mineral layer were multiplied by total soil dry weight:

$$\begin{array}{c} S\,o\,i\,l\,C\,p\,o\,o\,l\,s\,i\,z\,e={\left[C\right]}_{o\,r\,g\,a\,n\,i\,c}\times D\,W_{o\,r\,g\,a\,n\,i\,c}+{\left[C\right]}_{m\,i\,n\,e\,r\,a\,l}\times D\,W_{m\,i\,n\,e\,r\,a\,l}\\ S\,o\,i\,l\,N\,p\,o\,o\,l\,s\,i\,z\,e={\left[N\right]}_{o\,r\,g\,a\,n\,i\,c}\times D\,W_{o\,r\,g\,a\,n\,i\,c}+{\left[N\right]}_{m\,i\,n\,e\,r\,a\,l}\times D\,W_{m\,i\,n\,e\,r\,a\,l} \end{array}$$

Where *DW*_*organic*_ and *DW*_*mineral*_ are the area-based soil dry weight of the organic and mineral layers in the active layer. [C]*_*organic*_* and [C]*_*mineral*_* are the C concentrations, and [N]*_*organic*_* and [N]*_*mineral*_* are the N concentrations in the organic and mineral layers.

### Statistical Analysis

We calculated summary statistics of root traits and tested the significant effects of genera (alder, birch, willow, or sedge; vegetation effect) and sampling site location (local environment effect) with two-way ANOVA. Data were log-transformed to meet the normality requirement. We also performed a principal component analysis (PCA) with samples from all four genera plots or only plots dominated by shrubs. We included root δ^15^N in the PCAs as it indicates the dependence of N uptake on the ectomycorrhizal fungal pathway (Craine et al., 2015).

We then compared the plot-level root productivity, accumulative root biomass, and the soil C and N pool above the permafrost among plots with different dominant genera and sampling site locations with two-way ANOVA. Soil C was regressed against soil N among shrub-dominated and sedge-dominated plots, and the regression slopes were compared to evaluate impacts of dominant genus on the amount of C stored per unit N. Data were log-transformed to meet the normality requirement. We also compared the [N] in the soil POM component in the organic layer among plots with different dominant genera and sampling site locations. Finally, we calculated the difference between plant and organic layer soil δ^15^N values among dominant genus to evaluate the shrub impacts on the input and output of soil N. All statistics were performed using R (R Core Team., 2019; version 3.6.1; R Foundation for Statistical Computing; www.r-project.org). Data are expressed as mean ± standard error.

## Results

**FIGURE 2:**
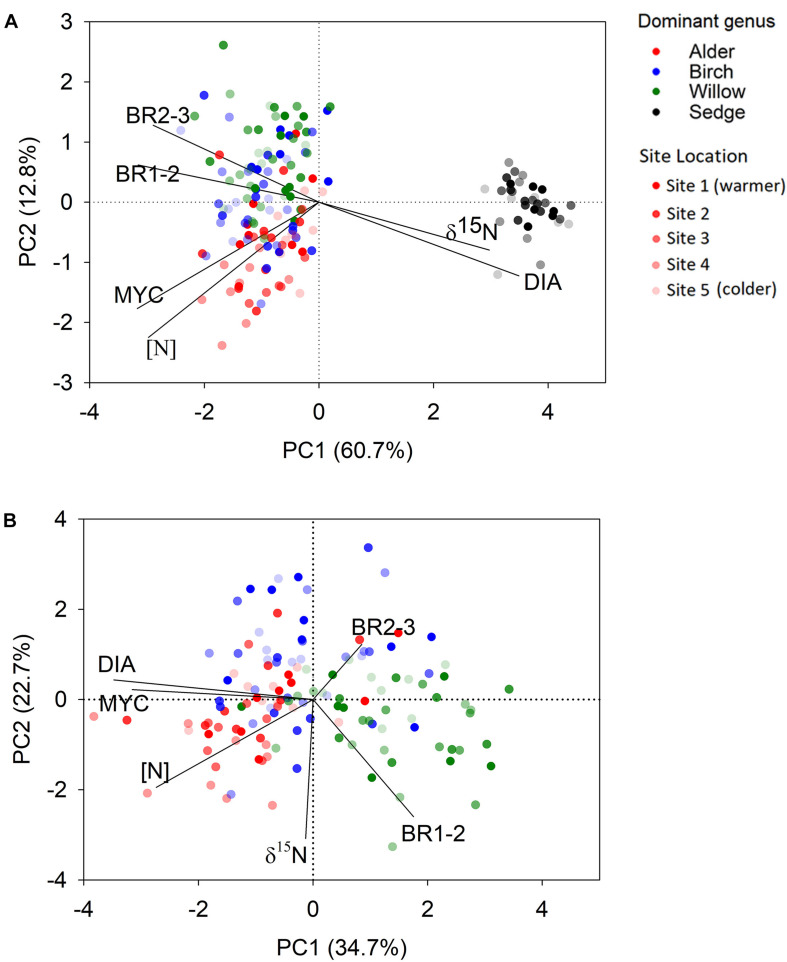
Score plot of shrubs and tussock sedges in the principal component analysis for all root samples **(A)** and shrub samples only **(B)**. Samples from higher latitude sites are indicated with greater transparency. DIA = root diameter, [N] = root nitrogen concentration, MYC = ectomycorrhizal colonization, BR1-2 = number of 1st order roots per each 2nd order root, BR2-3 = number of 2nd order roots per each 3rd order root, and δ^15^N = stable isotope ratios of nitrogen in roots. Statistical summary of the principal component analysis is shown in Supplementary Tables 3, 4.

We found striking differences in multiple traits between shrub and sedge roots (Table 2 and Figure 2A). PCA across all shrub and sedge samples identified only one significant axis (eigenvalue > 1) that explained 60.7% variations of the root trait dataset and separated the sedge root samples from those of the three shrub genera (Figure 2A). The primary component was associated with root diameter, branching ratio, root [N], root δ^15^N, and ectomycorrhizal colonization with similar loading scores (Supplementary Table 3). Among the three shrub genera, the absorptive roots of willow were generally thinner and branched more frequently than the roots of birch and alder. In contrast, alder and birch roots showed higher ectomycorrhizal colonization than willow (Table 2). The root [N] in alder was higher than the other two shrub genera (Table 2). PCA across shrub root samples identified three significant axes (eigenvalue > 1) of the variation in root traits. The primary component was associated with root diameter, root [N], and ectomycorrhizal colonization, and generally separated the willow samples from alder and birch (Figure 2B, Supplementary Table 4). The two-way ANOVA showed that the plant genera effect was significant on all root traits whereas the effect of site location was significant only on BR1-2 and root [N] among the root traits (Table 2).

**TABLE 2:** Root morphology, architecture, chemistry, and mycorrhizal colonization of four different vegetation types in Northern Alaska, United States.

	**Alder**	**Birch**	**Willow**	**Sedge**	**Dominant genus and site location effect**
Diameter (mm)	0.43 ± 0.01^a^	0.39 ± 0.01^a^	0.31 ± 0.01^b^	0.81 ± 0.02^c^	Dominant genus**, Site location^ns^, Interaction^ns^
BR1-2	4.4 ± 0.2^a^	4.3 ± 0.1^a^	5.7 ± 0.2^b^	1.0^c^	Dominant genus**, Site location*, Interaction^ns^
BR2-3	3.3 ± 0.15^a^	3.9 ± 0.23^ab^	4.1 ± 0.2^b^	1.0^c^	Dominant genus**, Site location^ns^, Interaction^ns^
[N] (%)	1.42 ± 0.05^a^	1.06 ± 0.03^b^	1.04 ± 0.03^b^	0.47 ± 0.03^c^	Dominant genus**, Site location**, Interaction^ns^
δ^15^N (‰)	−1.16 ± 0.18^a^	−2.62 ± 0.30^b^	−0.97 ± 0.20^a^	2.63 ± 0.14^c^	Dominant genus**, Site location^ns^, Interaction*
Ectomycorrhizas (%)	63.7 ± 2.4^a^	65.0 ± 2.9^a^	38.4 ± 3.2^b^	0^c^	Dominant genus**, Site location^ns^, Interaction*

Different lowercase letters indicate significant differences among the plots with different dominant genera (*P* < 0.05). Significances of dominant genus and site location effect are also shown (“**” for *P* < 0.001; “*” for *P* < 0.05; and “*ns*” for *P* > 0.05). Note: Absorptive root traits were averaged from individual measurements across the five sampling sites (*n* = 45 for each shrub genera and *n* = 35 for *Eriophorum*). BR1-2 is number of 1st order roots per each 2nd order root, and BR2-3 is number of 2nd order roots per each 3rd order root. Details are provided in section “*Materials and Methods*.”

**FIGURE 3:**
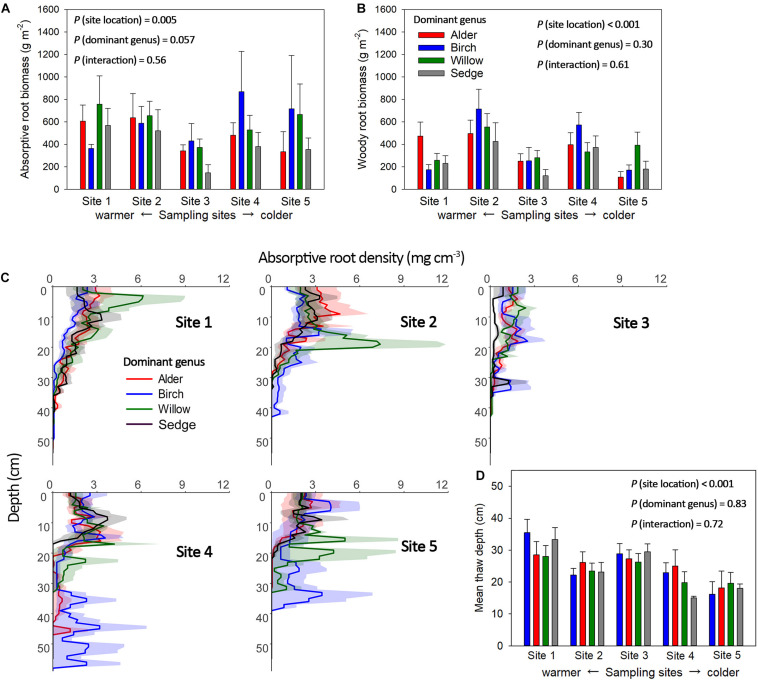
Accumulative absorptive **(A)** and woody **(B)** root biomass in the active soil layer, and vertical distribution of absorptive root biomass from the soil surface (moss excluded) to the top of the permafrost layer **(C)** of the plots with four different dominant genera across the five sampling sites. Mean thaw depth for plots with the same dominant genus at each site were also shown **(D)**. Shaded area and error bars indicate ± 1 S.E. Accumulative absorptive root biomass was significantly influenced by sampling site location (*P* = 0.005, Site 2 > Sites 3, 5; Site 1 > Site 3) and marginally influenced by dominant genus (*P* = 0.057). Woody root biomass was significantly influenced by only site location (*P* < 0.001, Site 2 > Sites 1, 3, 4, 5; Site 4 > Site 3). Mean thaw depth were significantly influenced by site location (*P* < 0.001, Site 1 > Sites 2, 4, 5; Site 3 > Sites 4, 5). There are more deep roots (> 20cm) in shrub (mainly birch) dominated plots than sedge dominated plots in Site 4 and 5 (*P* = 0.04).

**FIGURE 4:**
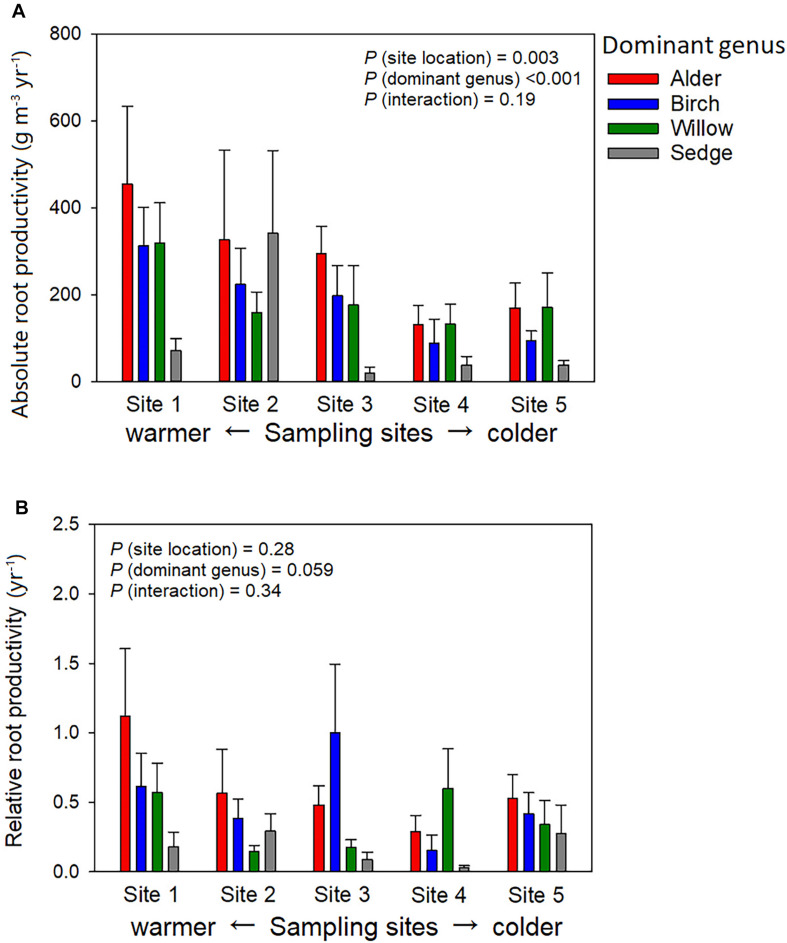
Community-level productivity of absorptive roots among four different vegetation types in absolute **(A)** and relative **(B)** manner. Absolute root productivity was the averaged biomass in the ingrowth cores, whereas relative root production was the ratio of absolute root production to the averaged initial root biomass before the root ingrowth experiment. Absolute root productivity was influenced by site location (*P* = 0.003, Sites 1, 2 > Site 4, Site 2 > Site 3) and dominant genus (*P* < 0.001, Alder, Birch, Willow > Sedge). Relative root productivity was only marginally influence by dominant genus (*P* = 0.059).

Absorptive roots accounted for 26.0 ± 2% of total root biomass on average across all plots, and the portion of absorptive roots did not significantly change with dominant genus or site location. Variation of active-layer accumulative absorptive root biomass of a plot was significantly influenced by sampling site location but not dominant genus (Figure 3A). In the two northern sites (Sites 4 and 5), several birch-dominated plots showed much greater absorptive root biomass than plots dominated by other genera in the deeper layers (>20 cm, Figure 3C). Non-absorptive (woody) root biomass was influenced by both site location and dominant genus, and we found considerable woody roots even in sedge-dominated plots (Figure 3B). Annual absolute root productivity tended to be higher in warmer sites than colder sites, and higher in the three shrub-dominated plots than sedge-dominated plots. Annual relative root productivity was only influenced by dominant genus, as sedge dominated plots showed smaller relative root productivity than the alder-dominated plots (Figure 4).

**FIGURE 5:**
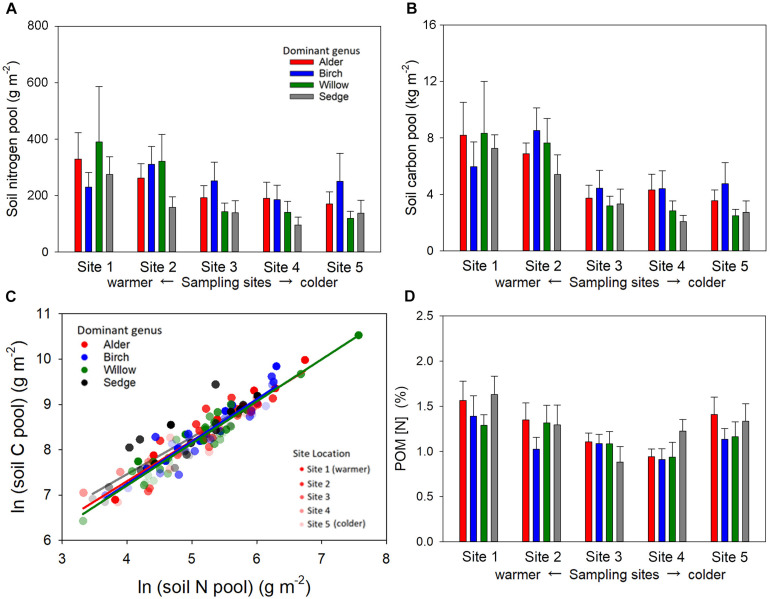
The pool size of soil nitrogen **(A)**, soil carbon **(B)**, and the relationship between log-transformed soil carbon and nitrogen **(C)** in the active soil layer, as well as [N] of particulate organic matter (POM) in the organic layer **(D)** of the shrub and sedge dominated plots across the five sampling sites. Both soil C and N pool were significantly influenced by site location (*P* < 0.001 for soil C, Sites 1, 2 > Sites 4, 5; Site 1 > Site 3; and *P* = 0.007 for soil N, Sites 1, 2 > Site 4, 5) but not dominant genus (*P* = 0.36 for soil C and *P* = 0.25 for soil N). Soil N was significantly correlated with soil C in both shrub and sedge dominated plots, but the slope of the regression did not significantly differ among plots with different dominant genera (*P* = 0.95). Samples from higher latitude sites are indicated with greater transparency in the scatter plot. The [N] of POM in the organic layer was sensitive to site location (*P* = 0.014, Site 1 > Sites 3, 4) but not dominant genus (*P* = 0.35).

Total C and N pools in the active layer soil were significantly influenced by sampling site location but not dominant genus (Figures 5A,B). Warmer sites showed greater soil C and N pools than colder sites. We did not find a difference in soil C per unit of soil N among the plots of different dominated genera (Figure 5C). The patterns of POM [N] in the organic layer among plots and sampling sites were similar to the patterns of total soil N pools (Figure 5D).

**FIGURE 6:**
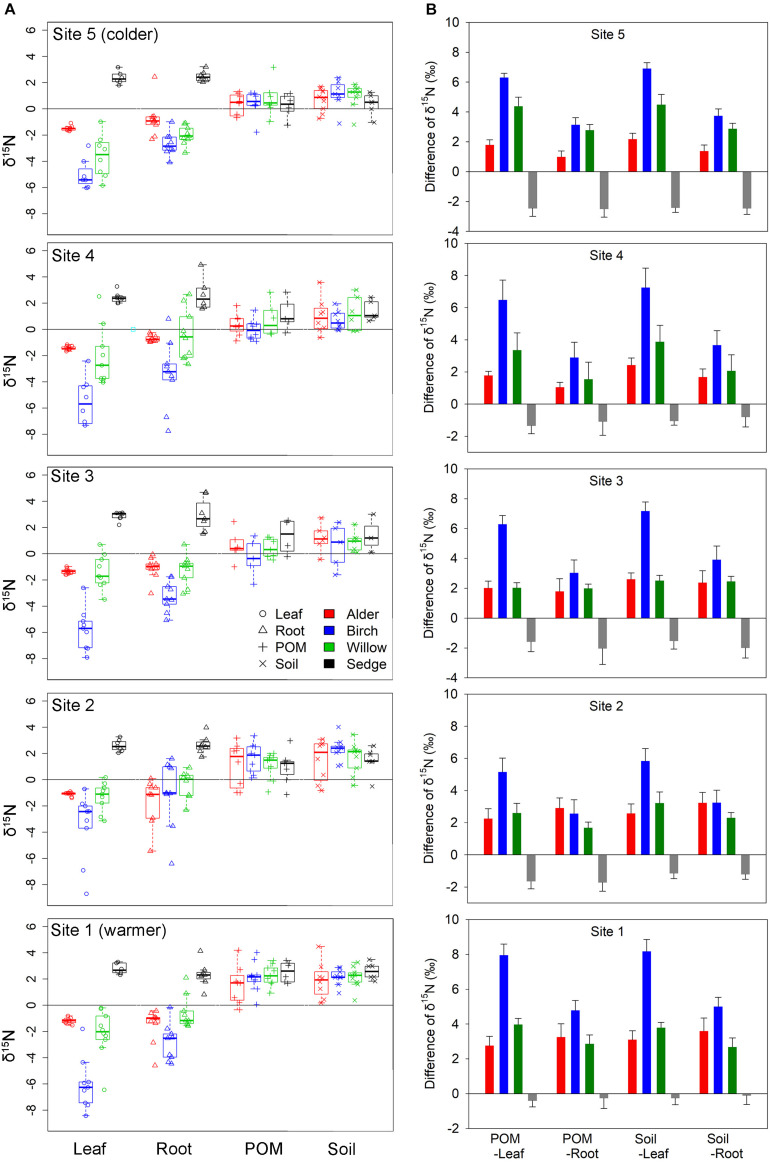
**(A)** The δ^15^N values of leaf, root, organic layer soil and its particulate organic matter (POM) component among plots with four different dominant genera at the five sites. Leaf and root δ^15^N were significantly influenced by dominant genus (*P* < 0.001, Sedge > Alder, Willow > Birch for both leaf and root) but not site location (*P* = 0.13 for leaf and 0.77 for root); soil and POM δ^15^N were significantly influenced by site location (*P* < 0.001, Site 1 > Sites 2, 3, 4, 5; Site 2 > Site 5 for both soil and POM) but not dominant genus (*P* = 0.89 for soil and 0.34 for POM); **(B)** Pair-wise comparison of soil δ^15^N (and POM component) vs. plant δ^15^N (leaf and root) among plots with four different dominant genera of the five sites. In shrub-dominated plots, plant δ^15^N (both leaf and root) was lower than the corresponding organic layer soil and POM δ^15^N, whereas in sedge-dominated plots the plant δ^15^N was higher than soil and POM δ^15^N. Effects of dominant genus on the difference in δ^15^N between soil and plant were significant across all sample sites (*P* < 0.001).

The plant δ^15^N values were generally negative of the three shrub genera, whereas δ^15^N values of sedge were generally positive (Figure 6A). Within the shrub group, birch has more negative δ^15^N values than willow and alder. Positive δ^15^N values were often found in the soil and its POM component in the organic layer, and did not differ among plots with different dominant genera (Figure 6A). In shrub-dominated plots, values of plant δ^15^N (both leaf and root) were lower than the organic layer soil and POM δ^15^N, whereas in sedge-dominated plots values of plant δ^15^N were higher than soil and POM δ^15^N (Figure 6B). The effects of dominant genus on the difference in δ^15^N between soil and plant were consistent across all sample sites (Figure 6B).

## Discussion

### Root Traits and Nutrient Acquisition Strategy

**FIGURE 7:**
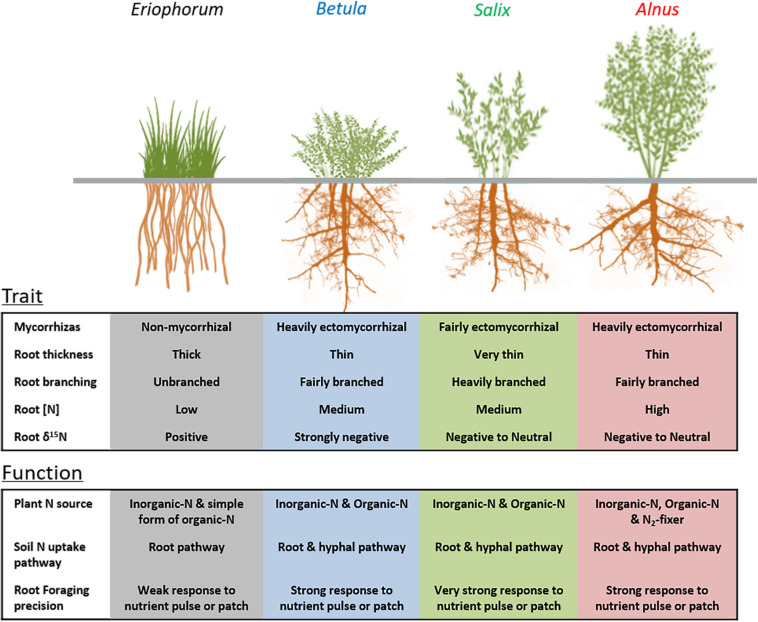
Diagram showing the differences in root traits and functions relevant to nutrient acquisition among arctic tundra shrubs (*Alnus*, *Salix*, and *Betula*) and sedges (*Eriophorum vaginatum*). Root functions are predicted by the root traits in this study and trait-function linkages reported in previous literature.

This study focused on the absorptive roots of arctic shrubs and sedges, which are the main portion of the root system serving the nutrient acquisition function. The consistent inter-genera differences in absorptive root traits along the sampling transect indicate contrasting root functions and nutrient acquisition strategies among the selected plant genera (Figure 7), irrespective of climatic and edaphic conditions. Among the shrub genera, the relatively lower efficiency in root length construction of alder and birch than willow has been compensated for by their higher ectomycorrhizal dependence, suggesting functional complementarity between shrub roots and ectomycorrhizal fungi in the arctic tundra (Chen et al., 2016; Bergmann et al., 2020). Also, small root diameter and intensive branching can create a large absorptive surface per unit root mass, which allows precise proliferation into ephemeral nutrient hotspots and rapid turnover of root material once nutrients are depleted in the hotspot (McCormack et al., 2012; Eissenstat et al., 2015; Chen et al., 2016; 2018). The variation of absorptive root diameter among shrub genera also suggests that it is problematic to use a universal diameter cutoff (e.g., 2 mm) across all arctic deciduous shrubs to compare “fine root” functional traits. Future studies that focus on absorptive portion of the arctic shrub root system and further identify the specific shrub genera in the tundra landscape will ultimately provide better predictions of belowground dynamics across the changing arctic tundra landscape. In addition, the large variation of root diameter between the three shrub genera and the sedge *E. vaginatum* might suggest distinct nutrient foraging strategies (Figure 7), but caution is needed in interpreting the diameter effect for a wider range of species than examined here.

The type of N source and N acquisition pathways also vary among the three shrub genera as indicated by their differences in δ^15^N. The root and leaf δ^15^N values are often more negative in birch than alder and willow. The relatively less negative δ^15^N of willow is in consistent with the lower ectomycorrhizal colonization, suggesting less dependence on the fungal hyphae in nutrient acquisition (Craine et al., 2015). On the other hand, the fixation of atmospheric N_2_ by alder causes the less negative δ^15^N values of alder leaves and roots than birch (Figure 6A; Peoples et al., 1989), despite their similar intensity of ectomycorrhizal colonization (Table 2). The nitrogen-fixing bacterium in alder root nodules can supply extra N to plants (Mitchell and Ruess, 2009). Salmon et al. (2019) found that N fixation supplies 16% of N required for annual net primary productivity of an alder shrubland in Western Alaska. The N-fixation probably causes the higher [N] in alder roots in our study, indicating stronger root metabolic activities of alder than birch and willow (Reich et al., 2008). Root nodules were detected in the alder root systems in all the five sites (Supplementary Figure 1), suggesting nitrogen fixations of alder across a wide range of climatic gradients in the arctic tundra. Overall, our findings of the unique root traits among the co-existing shrubs provide a better understanding of the fine-scale patterns of nutrient acquisition strategy and nutrient dynamics in the arctic tundra ecosystem.

### Absorptive Root Biomass and Distribution

At the community level, surface-layer absolute root productivity was strongly influenced by sampling site location and dominant genus. Moreover, among the plots dominated by different genera, absolute root productivity tended to be larger in shrub-dominated plots than in sedge-dominated plots at the time of sampling (early in the growing season). We also found that relative root productivity was marginally influenced by dominant genus (mainly between alder and sedge dominated plots), probably because of their large difference in root morphology and mycorrhizal type (Chen et al., 2018). However, we found that root biomass was not influenced by dominant genus, and root biomass was highly heterogeneous in the plots dominated by same the genera within a site (10.9-fold variation on average), indicating the importance of local factors other than plant genera in determining root growth (Valverde-Barrantes et al., 2015).

Active layer root biomass is mainly restricted by soil thaw depth in the early season since we found roots down to the bottom of the active layer in most of the plots, especially in two northern sites (Table 1 and Figure 3D). Interestingly, there are more deep roots (>20 cm depth) in some birch-dominated plots than sedge-dominated plots in the two northern sampling sites (*P* = 0.04), but not in other sites (Figure 3C). The birch crowns may trap more snow than sedges and create a stronger snow-insulation before the growing season (Sturm et al., 2001a), resulting in greater thaw depth under some of the birches than sedge. However, since roots are not entirely from the target genus in the plot, the reason for the variation in vertical root distribution among the three shrub genera is unclear, and could be the results of differences in allocation strategy, snow-insulation capacity and vegetation albedo among the shrubs (Sturm et al., 2001a; Chapin et al., 2005; Iversen et al., 2015). Future studies that sort roots into species using molecular barcoding techniques might better inform the species-specific allocation strategies (Hewitt et al., 2019b). In addition, the early-season patterns of rooting depth and vertical distribution of absorptive roots among the four dominant genera may shift as soil thaw continues in the mid-season. Nevertheless, the potentially warmer soil under some birch-dominated plots in the early season allows deeper proliferation and acquisition of nutrients by birch roots and associated ectomycorrhizal fungi in deeper layers (Hewitt et al., 2019a,b).

### Influence of Belowground Shrub Expansion on Soil Nitrogen

We predicted that the presence of woody detritus in shrub-dominated plots may decrease soil N per unit soil C, but this pattern did not exist in our sites (Figure 5C). Although C: N might be higher in the woody structures of the shrubs than herbaceous plants, the main N inputs from plants to soil were the leaf and absorptive roots that were lower in C: N in shrubs than sedges. Moreover, the variations in the tissue [N] of the dominant plant were not corresponding to variation in the POM [N] in the organic soil layer (Figure 5D), suggesting N resorption or immobilization of the shrub litter (Freschet et al., 2010b; McLaren et al., 2017). We also predicted that presence of ectomycorrhizal fungi in shrub -dominated plots might decrease soil N per unit soil C, but we found evidence that shrub roots are not rare into adjacent sedge-dominated plots, suggesting that ectomycorrhizal shrubs might have altered the N cycling in an area greater than their aboveground cover.

We found that the δ^15^N values of soils and POM in the organic layer of sedge-dominated plots were lower than the δ^15^N values of sedge leaves and roots (Figure 6B). Since decomposition of plant litter often results in enrichment of δ^15^N (Connin et al., 2001; Craine et al., 2015), one might expect higher δ^15^N values in soil and POM than in plant tissues, yet we found the exact opposite pattern in our sedge-dominated plots. One possible explanation is that there are N flows with more negative δ^15^N entering the sedge-dominated plots and lowering the average soil or POM δ^15^N. These potential N flows are probably due to the translocation of shrub leaf litter and/or turnover of encroaching shrub roots that are both strongly depleted in δ^15^N (Figure 6A). These potential N flows from shrub-dominated to sedge-dominated plots may also explain the limited variation in soil N pool and C: N ratio.

In summary, our study reveals profound variations in root traits, mycorrhizal symbioses and root functions relevant to nutrient acquisition among the widespread arctic plants (Figure 7). The variations of root traits are consistent along the climatic gradient in the arctic tundra, portending widespread belowground trait shifts across regions experiencing changes of aboveground vegetation (e.g., shrub expansion). In addition, we found that shrub-dominated plots tended to have more productive absorptive roots than sedge-dominated plots, and the patterns of root vertical distributions vary among plots with different dominant shrub genera. However, the pool of soil N is not influenced by the genus that was dominant aboveground and generally decreased from warmer to colder sites. More importantly, we found large variations in absorptive root traits, root productivity and biomass, as well as soil nutrient dynamics across our sampling area in northern Alaska, suggesting that future studies across wider geographical ranges and broader plant lineages are urgently needed for a more holistic understanding of belowground nutrient processes and to improve predictions of nutrient dynamics across the changing Arctic.

## Data Availability Statement

The datasets presented in this study can be found in online repositories. The names of the repository/repositories and accession number(s) can be found below: The data used in this study are available on ESS-DIVE: Fraterrigo, J., and W. Chen. 2020. Arctic shrub root traits, northern Alaska, summer 2017. Arctic Shrub Expansion, Plant Functional Trait Variation, and Effects on Belowground Carbon Cycling. doi: 10.15485/1631262.

## Author Contributions

JF conceived the study. WC, KT, EE, and JF designed the study. WC, KT, and JF collected the data. WC and SL analyzed the data. AM and JG contributed computer code. WC wrote the manuscript, with input from KT, SL, EE, and JF. All authors contributed to the article and approved the submitted version.

## Conflict of Interest

The authors declare that the research was conducted in the absence of any commercial or financial relationships that could be construed as a potential conflict of interest.
