# Supplementary material for: Impacts of Arctic Shrubs on Root Traits and Belowground Nutrient Cycles Across a Northern Alaskan Climate Gradient
Source: Front Plant Sci. 2020 Dec 11;11:588098. doi: 10.3389/fpls.2020.588098 (PMC7758488; doi:10.3389/fpls.2020.588098)
Supplement: Supplementary file 1 [file Table_1.DOCX]

**Supplementary**

**Impacts of arctic shrubs on root traits and belowground nutrient cycles across a Northern Alaskan climate gradient**

Weile Chen, Ken D. Tape, Eugénie S. Euskirchen, Shuang Liang, Adriano Matos, Jonathan Greenberg, Jennifer M. Fraterrigo

**Supplementary Figures and Tables**


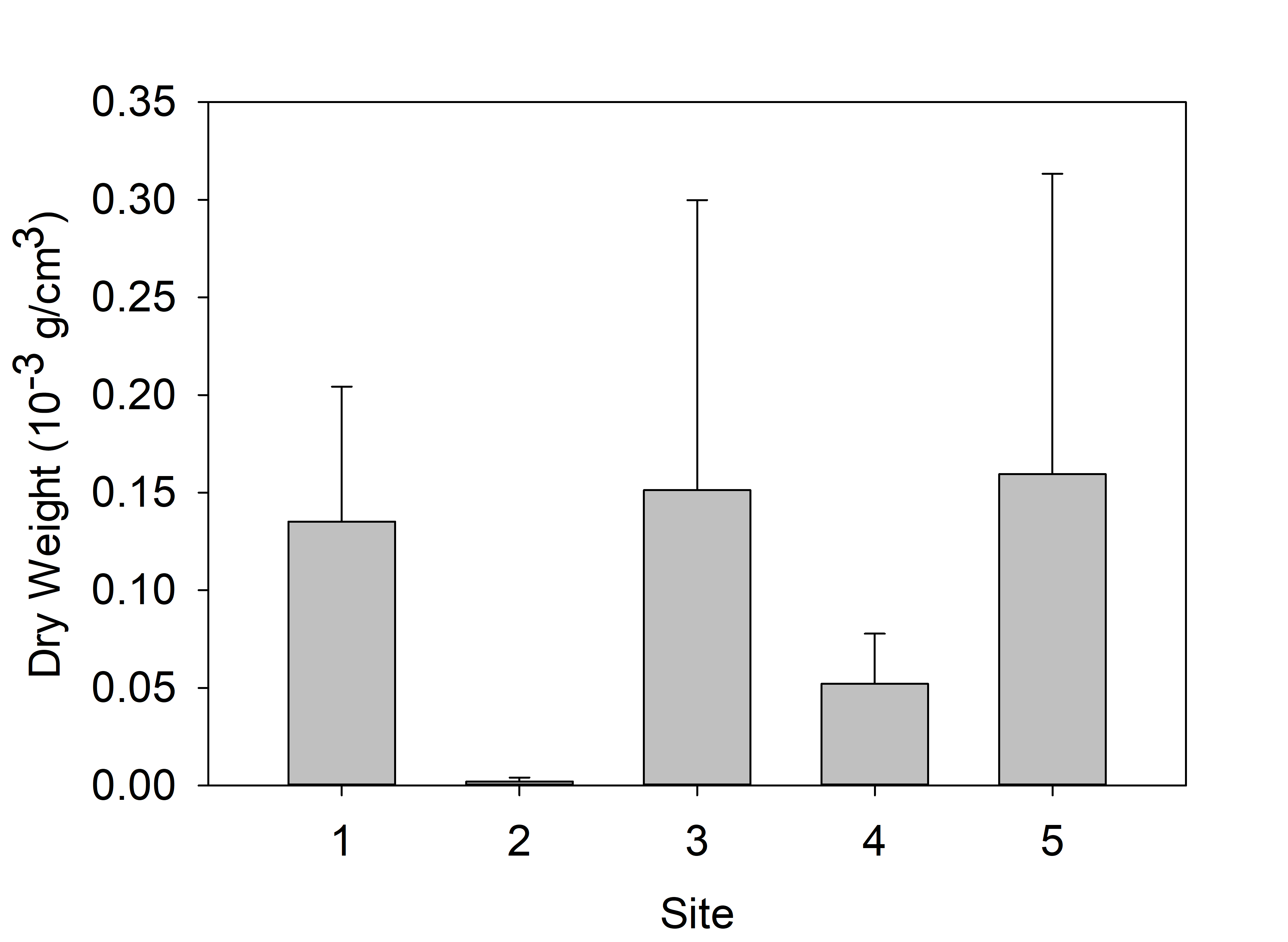


Figure S1. Nodule dry weight in alder plots in each sampling site. Nodules were collected in four random soil cores (3.5-cm diameter, 10-cm deep) in each plot in late-June to early-July, 2017.

Table S1. Historical climatic variables of the five sampling sites. Data was obtained from the Scenarios Network for Alaska & Arctic Planning (SNAP). Annual air temperature was averaged from 1985-2015, and other variables were averaged from 2000-2009.

|  | Air temperature | | | Annual precipitation^4^ | Growing season length^5^ |
| --- | --- | --- | --- | --- | --- |
|  | Annual^1^ | Summer^2^ | Winter^3^ |  |  |
| Site 1 | -6.6 | 13.4 | -20.6 | 441 | 154 |
| Site 2 | -6.0 | 13.6 | -19.8 | 294 | 154 |
| Site 3 | -7.4 | 12.6 | -20.8 | 252 | 148 |
| Site 4 | -9.0 | 10.4 | -22.6 | 192 | 126 |
| Site 5 | -9.5 | 10.1 | -24.0 | 200 | 123 |

^1^http://ckan.snap.uaf.edu/dataset/historical-monthly-and-derived-temperature-products-771m-cru-ts

^2^**(June-August)** http://ckan.snap.uaf.edu/dataset/historical-monthly-and-derived-temperature-products-771m-cru-ts

^3^**(December-February)** <http://ckan.snap.uaf.edu/dataset/historical-monthly-and-derived-temperature-products-771m-cru-ts>

^4^http://ckan.snap.uaf.edu/dataset/historical-monthly-and-derived-precipitation-products-771m-cru-ts

^5^http://ckan.snap.uaf.edu/dataset/historical-derived-dof-dot-logs-771m-cru-ts

Table S2. Species list of *Salix* (willow) in each sampling site. Willow species’ relative abundance in each site is also shown.

| Site | *Salix* species |
| --- | --- |
| 1 | *S. glauca* (11%), *S. pulchra* (89%) |
| 2 | *S. glauca* (44%), *S. pulchra* (56%) |
| 3 | *S. glauca* (78%), *S. pulchra* (11%), *S. arbuscoloides* (11%) |
| 4 | *S. pulchra* (100%) |
| 5 | *S. glauca* (67%), *S. pulchra* (11%), *S. richarosonii* (22%) |

Table S3. Eigenvalues of the first two components from the principal component analysis of all shrub and sedge root samples (top table) and loading scores of root traits on each component (bottom table).

| Component | Eigenvalue | Proportion |
| --- | --- | --- |
| 1 | 3.64 | 60.7% |
| 2 | 0.77 | 12.8% |

| Variable | Component 1 | Component 2 |
| --- | --- | --- |
| Diameter | 0.46 | -0.35 |
| BR1-2 | -0.41 | 0.18 |
| BR2-3 | -0.38 | 0.36 |
| [N] | -0.39 | -0.64 |
| δ^15^N | 0.39 | -0.23 |
| Mycorrhizas | -0.41 | -0.50 |

Note: BR1-2 is number of 1^st^ order roots per each 2^nd^ order root, and BR2-3 is number of 2^nd^ order roots per each 3^rd^ order root. Details are provided in *Materials and Methods*.

Table S4. Eigenvalues of the three significant components (eigenvalue >1) from the principal component analysis of only shrub root samples (top table) and loading scores of root traits on each component (bottom table).

| Component | Eigenvalue | Proportion |
| --- | --- | --- |
| 1 | 2.08 | 34.7% |
| 2 | 1.36 | 22.7% |
| 3 | 1.05 | 17.4% |

| Variable | Component 1 | Component 2 | Component 3 |
| --- | --- | --- | --- |
| Diameter | -0.87 | 0.11 | -0.05 |
| BR1-2 | 0.44 | -0.65 | -0.24 |
| BR2-3 | 0.21 | 0.31 | 0.87 |
| [N] | -0.68 | -0.49 | 0.12 |
| δ^15^N | -0.03 | -0.77 | 0.47 |
| Mycorrhizas | -0.79 | 0.06 | 0.03 |

Note: BR1-2 is number of 1^st^ order roots per each 2^nd^ order root, and BR2-3 is number of 2^nd^ order roots per each 3^rd^ order root. Details are provided in *Materials and Methods*.
